# Prenatal Alcohol Exposure and Cellular Differentiation: A Role for Polycomb and Trithorax Group Proteins in FAS Phenotypes?

**DOI:** 10.35946/arcr.v35.1.09

**Published:** 2013

**Authors:** Kylee J. Veazey, Daria Muller, Michael C. Golding

**Affiliations:** **Kylee J. Veazey***is graduate assistant, in the Department of Veterinary Physiology and Pharmacology, College of Veterinary Medicine and Biomedical Sciences, Texas A&M University, College Station, Texas.*; **Daria Muller***is graduate assistant, in the Department of Veterinary Physiology and Pharmacology, College of Veterinary Medicine and Biomedical Sciences, Texas A&M University, College Station, Texas.*; **Michael C. Golding, Ph.D.,***is an assistant professor, in the Department of Veterinary Physiology and Pharmacology, College of Veterinary Medicine and Biomedical Sciences, Texas A&M University, College Station, Texas.*

**Keywords:** Alcohol exposure, ethanol exposure, prenatal alcohol exposure, prenatal alcohol exposure, fetal alcohol spectrum disorders, fetal alcohol syndrome (FAS), FAS phenotypes, fetal development, epigenetics, epigenetic mechanisms, epigenetic changes, gene expression, developmental programming, transcription, cellular differentiation, Polycomb group proteins, Trithorax group proteins

## Abstract

Exposure to alcohol significantly alters the developmental trajectory of progenitor cells and fundamentally compromises tissue formation (i.e., histogenesis). Emerging research suggests that ethanol can impair mammalian development by interfering with the execution of molecular programs governing differentiation. For example, ethanol exposure disrupts cellular migration, changes cell–cell interactions, and alters growth factor signaling pathways. Additionally, ethanol can alter epigenetic mechanisms controlling gene expression. Normally, lineage-specific regulatory factors (i.e., transcription factors) establish the transcriptional networks of each new cell type; the cell’s identity then is maintained through epigenetic alterations in the way in which the DNA encoding each gene becomes packaged within the chromatin. Ethanol exposure can induce epigenetic changes that do not induce genetic mutations but nonetheless alter the course of fetal development and result in a large array of patterning defects. Two crucial enzyme complexes—the Polycomb and Trithorax proteins—are central to the epigenetic programs controlling the intricate balance between self-renewal and the execution of cellular differentiation, with diametrically opposed functions. Prenatal ethanol exposure may disrupt the functions of these two enzyme complexes, altering a crucial aspect of mammalian differentiation. Characterizing the involvement of Polycomb and Trithorax group complexes in the etiology of fetal alcohol spectrum disorders will undoubtedly enhance understanding of the role that epigenetic programming plays in this complex disorder.

Exposure of the developing embryo and fetus to alcohol can have profound adverse effects on physical, behavioral, and cognitive development. The resulting deficits collectively have been termed fetal alcohol spectrum disorders (FASD). They range in severity from mild cognitive deficits to a well-defined syndrome (i.e., fetal alcohol syndrome [FAS]), which is broadly characterized by low birth weight, distinctive craniofacial malformations, smaller-than-normal head size (i.e., microcephaly), and central nervous system dysfunction (Riley et al. 2011). The mechanisms underlying ethanol’s harmful effects on development are not yet fully understood. Studies in recent years have indicated that epigenetic mechanisms may play a role in the etiology of FASD. This article describes the proposed roles of epigenetic mechanisms in FASD and cell differentiation in general and introduces two protein complexes that are hypothesized to play central roles in these events.

## Role of Epigenetics in Developmental Programming and FASD

Mammalian development consists of a series of carefully orchestrated changes in gene expression that occur as stem or progenitor cells differentiate to form the tissues and organs making up the growing fetus.^1^ Once the identity of each new cell type has been established by lineage-specific transcription factors, this identity is maintained through unique alterations in the way in which the DNA encoding each gene becomes packaged around certain proteins (i.e., the histones) within the chromatin structure of the nucleus (Hemberger et al. 2009). Much like a closed book cannot be read whereas an open book can, the DNA can either be tightly wound up into a structure that silences the encoded genes, or the DNA can be in a relaxed, open, and active state. As development proceeds, the DNA of each cell becomes packaged in a way that is unique to that cell type and thus is programmed to express only a specific set of genes that confer the cell’s individual identity and physiological function (Barrero et al. 2010). Three enzymatic mechanisms control the assembly and regulation of chromatin structure: DNA methylation, modification of the histone proteins (i.e., posttranslational histone modification), and ATP-dependent chromatin remodeling (Barrero et al. 2010). These fundamental modifications, which control gene packaging, are passed on to the daughter cells when a cell divides. They are referred to as epigenetic changes because they impart a level of regulation that is above (“epi”) the direct genetic modifications of the DNA (Hemberger et al. 2009).

Studies using a diverse range of model organisms have led to the conclusion that epigenetic modifications to the chromatin structure provide a plausible link between exposure to environmental substances that can harm the developing fetus (i.e., teratogens) and lasting alterations in gene expression leading to disease phenotypes. Numerous studies have demonstrated that exposure to ethanol is associated with both genome-wide and gene-specific changes in DNA methylation (Bielawski et al. 2002; Downing et al. 2011; Garro et al. 1991; Haycock and Ramsey 2009; Hicks et al. 2010; Liu et al. 2009; Ouko et al. 2009; Zhou et al. 2011), alterations in posttranslational histone modifications (Kim and Shukla 2005; Pal-Bhadra et al. 2007; Park et al. 2005), and a profound shift in epigenetically sensitive phenotypes (Kaminen-Ahola et al. 2010). Collectively, all of these observations indicate that ethanol can act as a powerful epigenetic disruptor and alter chromatin structure.

Although the mechanisms by which alcohol impacts chromatin structure are not completely understood, recent work suggests that some epigenetic changes result from altered cellular metabolism. For example, Choudhury and colleagues (2010) observed an increase in reactive oxygen species (ROS) within primary rat liver cells (i.e., hepatocytes) treated with ethanol. This increase in ROS was correlated with an increase in a specific modification of histone 3 (i.e., acetylation of histone 3 at lysine 9); moreover, when the cells were treated with cellular antioxidants to eliminate the ROS, these alcohol-induced chromatin modifications were abated (Choudhury et al. 2010). In addition, ethanol exposure has well-documented effects on one-carbon metabolism and the bioavailability of the crucial methyl donor, s-adenosylmethionine (SAMe). Impaired levels of SAMe disrupt the cells’ ability to methylate DNA and histones, resulting in compromised epigenetic programming (Zeisel 2011). Interestingly, many of the birth defects observed in FASD also have been noted in studies examining deficiencies in one-carbon metabolism (summarized in Zeisel 2011).

Although alcohol induces several global changes in chromatin structure, many of the associated developmental defects seem to be rooted in gene-specific alterations. A study by Hashimoto-Torii and colleagues (2011) examining global changes in gene transcription within ethanol-exposed samples of brain tissue (i.e., cerebral cortex) reported that of 39,000 candidate messenger RNAs (mRNAs) assessed, only 636 transcripts were differentially expressed. Other researchers have identified alcohol-induced alterations in the expression of only a small number of key developmental regulators, including several transcription factors known as *HOX* factors, which play crucial roles in directing organ patterning and morphogenesis (Godin et al. 2011; Mo et al. 2012; Rifas et al. 1997; Vangipuram and Lyman 2012). In rodent models, these alterations have been associated with neural patterning defects and the development of abnormalities in structures of the head and face (i.e., craniofacial dysmorphogenesis), reminiscent of those observed in clinical studies of FASD (Parnell et al. 2009; Rifas et al. 1997). However, these alcohol-induced alterations in gene expression often are limited to a specific tissue type and arise only when ethanol exposure occurs during select developmental windows (Godin et al. 2011; Kim et al. 2010; Mo et al. 2012; Parnell et al. 2009). These observations suggest that the molecular machinery involved in epigenetic programming also may be disrupted by ethanol exposure and, as a consequence, key epigenetic cues regulating development are not properly established.

## Epigenetic Control and Developmental Programming of Differentiation

Of the three classes of epigenetic modifications, posttranslational modification of histone proteins undoubtedly is the most complex. Posttranslational enzymatic modifications, such as acetylation, methylation, phosphorylation, and ubiquitination (which have been studied most extensively), work together to produce a combinatorial “histone code” that serves to regulate cell-lineage– specific patterns of chromatin structure throughout development (Fisher and Fisher 2011). Within the unique transcriptional environment of embryonic stem cells, several developmentally crucial genes are marked in a coordinated fashion with both activating and repressive histone modifications (Bernstein et al. 2006; Jiang et al. 2011; Lim et al. 2009). Specifically, histone 3 (around which DNA sequences are wrapped) is modified by the addition of three methyl groups to the fourth lysine residue (i.e., histone 3 lysine 4 trimethylation), which typically is associated with gene activation, as well as by trimethylation of lysine 27, which has repressive effects (see figure 1A). The DNA sequences wrapped around these uniquely marked histones are termed bivalent domains and generally encode transcription factors directing tissue-specific programs of differentiation (Fisher and Fischer 2011). This same distinctive signature is found, albeit less frequently, in placental, neuronal, and other tissue-specific progenitor cell types (Lim et al. 2009; Rugg-Gunn et al. 2010). These bivalently marked genes generally are not expressed but are thought to be “primed” for either rapid activation or silencing during differentiation. Once a progenitor cell’s fate has been established by lineage-specific transcription factor networks, the cell’s transcriptional memory is maintained by removing one of the coexisting modifications and leaving only the modification indicative of the active or silent state in place. Importantly, many bivalently marked genes are disrupted in prenatal models of alcohol exposure, which potentially may explain the constellation of effects observed in FASD. For example, in a neural stem cell model ethanol exposure alters both histone 3 lysine 4 and lysine 27 trimethylation (Veazey et al. 2013). Understanding the mechanistic basis of these epigenetic defects is crucial to deciphering the developmental origins of FASD.

Seminal studies using the fruit fly *Drosophila melanogaster* in the late 1970s to early 1980s revealed the existence of two large multiprotein complexes with diametrically opposite roles in the regulation of gene expression: the Polycomb group (PcG) and Trithorax group (TrxG) (Lewis 1978; Poux et al. 2002; Schuettengruber et al. 2007). These two developmentally crucial enzyme complexes function at the hub of mammalian development; by binding to the regulatory regions of bivalent genes, they regulate the intricate balance between self-renewal of stem and progenitor cells and the execution of cellular differentiation. As differentiation progresses, these regulatory regions “commit” to one of these two protein complexes and become occupied exclusively by either the PcG or TrxG proteins. This commitment occurs in a cell-lineage–dependent manner, and as a result the chromatin structure of these bivalent genes becomes fixed in either an active or a silent state. Any defects in this delicate balancing act, particularly during the differentiation towards a neural lineage, results in developmental defects and causes disease. Despite their fundamental importance to the processes of epigenetic programming and mammalian development, however, the roles of PcG and TrxG proteins in the etiology of FASD to date have not been examined.

## PcG Proteins

**Figure 2A:**
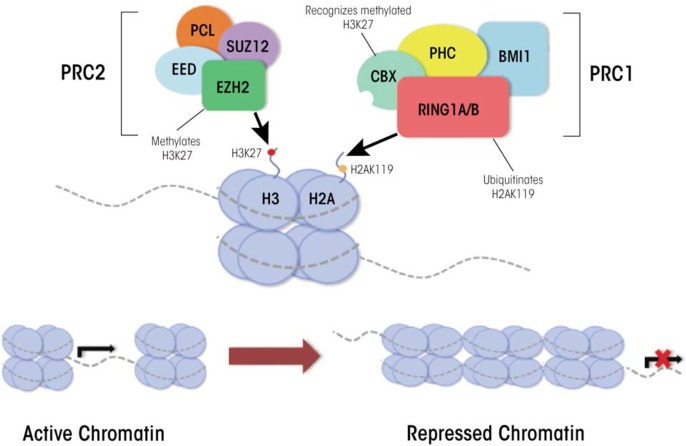
Transcriptional regulation by the Polycomb and Trithorax complexes. Polycomb repressive complex 1 (PRC1) consists of four core proteins including: polyhomeotic homolog (PHC), ring finger protein 1A or 1B (RING1A or RING1B), B-lymphoma Mo-MLV insertion region 1 homolog (BMI1), and chromobox homolog (CBX). The RING1A/RING1B subunits are the catalytic engine of the PRC1 complex and carry out ubiquitination of histone 2A at lysine 119 (H2AK119ub). PRC2 consists of four core proteins including: embryonic ectoderm development (EED), enhancer of zeste 2 (EZH2), suppressor of zeste 12 (SUZ12), and polycomb like (PCL). EZH2 serves as the catalytic subunit of PRC2 and trimethylates lysine 27 on histone 3 (H3K27me3). Current models suggest that H3K27me3 generated by PRC2 facilitates compaction of chromatin leading to the repression of gene expression. Subsequently, the CBX subunit of the PRC1 complex recognizes H3K27me3, and the RING1A/RING1B subunits of PRC1 ubiquitinate H2AK119 to facilitate the maintenance of the repressed state.

The PcG proteins and the genes encoding them originally were discovered over 30 years ago as key regulators of the processes that specify which end of the embryo forms the head and which the rear during the development of Drosophila (Lewis 1978). Since then, researchers have found that these gene families encode essential regulators governing mammalian processes of cellular determination and lineage-specific patterns of differentiation. In mammals, two major PcG complexes have been characterized that modify chromatin structure; these are called Polycomb Repressive Complexes 1 and 2 (PRC1 and PRC2). Each complex is composed of several proteins with different biochemical functions, many of which are not well understood (see figure 2). PRC1 acts by mediating the ubiquitination of the 119th lysine residue of histone H2A; this is achieved by two of the PRC1 proteins called ring finger protein 1A and 1B (RING1A and RING1B) (Wang et al. 2004). This posttranslational modification pushes the local chromatin structure towards a transcriptionally repressive state and its proper establishment is essential to the coordinated silencing of genes through-out mammalian development (Boyer et al. 2006; Wang et al. 2004). In embryonic stem cells, histone ubiquitination stabilizes the presence of an enzyme called RNA polymerase II (which is required for gene expression) at bivalent chromatin domains and is crucial for maintaining the pluripotent state of undifferentiated cells (Ku et al. 2008).

PRC2 has similar repressive properties to PRC1 and also is an essential regulator of cellular differentiation. It facilitates the silencing of developmentally crucial genes through mono-, di-, and trimethylation of the histone 3 lysine 27 and trimethylation of histone 3 lysine 9 (Cao et al. 2002; Czermin et al. 2002), both of which repress gene expression. Together, the methylation of these two lysine residues promotes the generation of facultative heterochromatin^2^ and mediates a transcriptionally silent state.

Adding an additional layer of complexity, PRC2 associates with the mammalian enzymes responsible for DNA methylation (i.e., DNA methyltransferase complexes); this association aids in the ability of PRC2 complexes to repress their target loci (Viré et al. 2006). This physical interaction suggests that the PcG complexes and the DNA methyltransferases act together to maintain the epigenetic memory of chromatin states throughout differentiation. Proper functioning of this gene family and their interacting proteins is essential for the execution of cell-specific differentiation programs and proper lineage specification (Pasini et al. 2007).

## TrxG Proteins

In fruit flies, maternal transcription factors that were included in the egg cells and which are distributed unevenly throughout the developing embryo shape gene expression in the early embryo. The levels of these transcription factors diminish over time, and once they disappear from the developing embryo, the memory of which genes were active in a given cell is propagated through the action of the TrxG proteins (Lewis 1978; Poux et al. 2002). These proteins also have been identified in mammals, where they have been implicated in fundamental epigenetic and cellular processes, including X-chromosome inactivation, genomic imprinting, stress response, programmed cell death (i.e., apoptosis), development of tumors (i.e., tumorigenesis), cell proliferation, and embryonic stem cell renewal. However, compared with the PRC1 and PRC2 complexes, very little information exists on individual TrxG proteins or their biochemical functions (Schuettengruber et al. 2007). It is known that TrxG proteins function as multiprotein complexes that mediate the trimethylation of histone 3 lysine 4 (H3K4me3) and which have been conserved across different species (Jiang et al. 2011). In mammalian cells the TrxG complex is formed by a core group of structural proteins that combine with at least one of six interchangeable histone methyltransferases.

**Figure 3:**
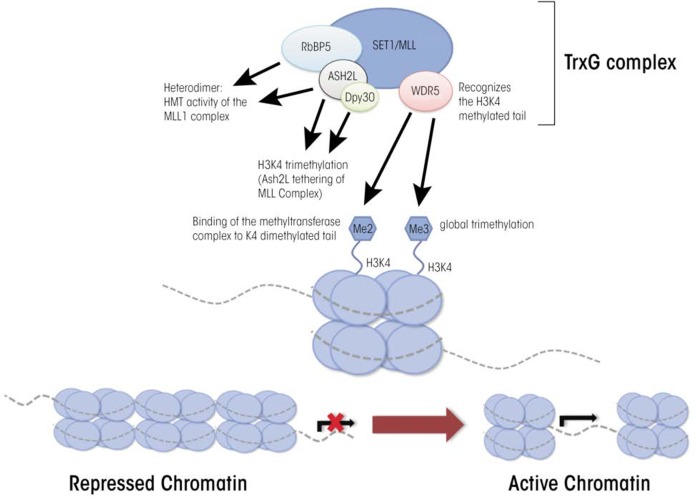
Trithorax (TrxG) proteins function as a conserved multi-component complex that regulates the trimethylation of histone 3 lysine 4 (H3K4me3). The four core structural components of the TrxG complex are: WD40 repeat domain 5 (WDR5), retinoblastoma binding protein 5 (RbBP5), dosage compensation-related protein 30 (Dpy30), and absent, small, or homeotic-like (Ash2L). These proteins serve as a scaffold to regulate the biological activity of the H3K4 methyltransferase family of enzymes, which include mixed-lineage leukemia (MLL) proteins MLL1, MLL2, MLL3, and MLL4, as well as SET1A and SET1B.

The main core of TrxG complexes is composed of four proteins called WD40 repeat domain 5 (WDR5), retinoblastoma binding protein 5 (RbBP5), dosage compensation–related protein 30 (Dpy30), and absent, small, or homeotic-like protein (Ash2L) (see figure 3). WDR5 recognizes histone 3 molecules that are methylated at lysine 4 and allows the methyltransferase in the TrxG complex to bind to this region and add another methyl group; thus, WDR5 is an essential regulator of global H3K4 trimethylation (Wysocka et al. 2005). RbB5 is necessary for proper differentiation of embryonic stem cells into neural progenitor cells and, together with Dpy30, also is essential for regulating global levels of H3K4 trimethylation (Jiang et al. 2011).

The TrxG core interacts with a group of interchangeable H3K4 methyltransferases, including some called mixed lineage leukemia (MLL) proteins (i.e., MLL1, MLL2, MLL3, and MLL4) and proteins called SET1A and SET1B (Jiang et al. 2011; Steward et al. 2006). MLL1 initially was discovered in cells of patients with different types of leukemia (i.e., acute lymphoid and acute myeloid leukemia). It is thought to promote cell-specific patterns of gene expression by regulating global and gene-specific H3K4 methylation during early embryonic development (Yu et al. 1995), because mice in which the corresponding mouse gene (*MLL1*) has been eliminated, or knocked out, show alterations in H3K4 methylation. In contrast, knockout of the *MLL2* gene in mouse embryonic stem cells leads to skewed differentiation but no concrete alterations to H3K4 methylation (Lubitz et al. 2007). For the remaining methyltransferases (i.e., MLL3, MLL4, and SET1A/1B), little is known except that they are involved in H3K4 methylation. Deletion of any one of these other methyltransferases seems to have only minimal effects on global levels of H3K4 methylation, likely because the remaining MLL family members can substitute for the deleted ones (Jiang et al. 2011). Thus, although researchers have made progress in clarifying the roles of TrxG proteins, much remains unknown regarding the temporal and tissue-specific regulatory events these proteins promote.

## Role of PcG and TrxG in the Etiology of FASD

Postmortem studies of children that succumbed to FAS revealed groups of poorly differentiated neuronal and glial cells at abnormal sites within the brain, suggesting large-scale problems with cellular proliferation and differentiation resulting from prenatal alcohol exposure (Swayze et al. 1997). Furthermore, studies using animal models have demonstrated reduced brain size and abnormal migration of neural cells in mice exposed to ethanol in utero (Godin et al. 2010; Parnell et al. 2009). Collectively, these observations indicate that alcohol impairs the cellular processes of neuronal differentiation and migration during fetal development. In support of this conclusion, studies using human and rodent neurosphere cultures have demonstrated that treatment with ethanol increases neurosphere size, skews the developmental potential of neural progenitor cells, and fundamentally alters the neuronal differentiation program (Roitbak et al. 2011; Vangipuram and Lyman 2012). However, the specific molecular mechanisms by which ethanol disrupts the cellular processes governing differentiation remain poorly defined. Recent studies examining the consequences of ethanol exposure during embryonic stem cell differentiation demonstrate a delay in the ability of exposed cells to silence regulatory factors promoting pluripotency, including the transcription factors OCT4, NANOG, and SOX2 (Arzumnayan et al. 2009). These studies strongly suggest that ethanol interferes with the ability of differentiating cells to recruit epigenetic modifiers to genes playing key roles in development and to execute the molecular programs governing cellular differentiation.

During early mammalian development, approximately 2,000 genes are bivalently marked as described earlier, and these marks progressively resolve towards the lineage-specific patterns of chromatin organization characterizing each unique cell type (Rugg-Gunn et al. 2010). As development proceeds, many precursor cell types maintain a subset of developmentally critical genes in this conformation as well push new groups of cellular factors into a bivalent state. For example, in pluripotent embryonic stem cells (which can differentiate into any cell type) the neural precursor genes *Dlx2, Hand1, Msx2, Nestin, Nkx2.1, Nkx2.2, Olig2, Pax6*, and *Sox1* all are bivalently marked, whereas in multipotent, neural precursor cells (which only can develop further into different types of neurons) only *Dlx2* and *Pax6* maintain this conformation. Interestingly, two genes encoding marker proteins that are found only in a type of glial cell called astrocyte (i.e., myelin basic protein [MBP] and glial fibrillary acidic protein [GFAP]) establish novel bivalent domains so that these genes can be kept in an active or inactive state, depending on whether they will become nerve cells or astrocytes (Golebiewska et al. 2009). Proper functioning of the TrxG complexes is indispensable to converting these bivalent loci into the actively transcribed state required for the induction of nerve cell formation (i.e., neurogenesis) (Huang et al. 2007; Jiang et al. 2011; Lim et al. 2009). Similarly, PcG complexes are necessary to silence the myriad of developmental regulators that would be required if the cells would differentiate into other cell types; thus, these complexes also help ensure that lineage-specific patterns of gene expression arise (Pereira et al. 2010). By propagating the transcriptional memory established by lineage-specific transcription factor networks, the TrxG and PcG complexes cooperatively regulate the balance between stem cell renewal and lineage differentiation.

Importantly, the expression of many of these factors that are bivalently marked and regulated by PcG and TrxG is disrupted in various models of prenatal alcohol exposure; moreover, this disruption is associated with profound errors in neuronal patterning. For example, alcohol suppresses the activation of two neural precursor genes—*Msx2* and *Pax6*—leading to craniofacial abnormalities and excessive differentiation of glutamatergic neurons, respectively (Kim et al. 2010; Mo et al. 2012; Rifas et al. 1997). Similarly, both the expression and localization of *Nkx2.1* and *Olig2* are diminished by alcohol, potentially disrupting the balance between excitation and inhibition in the cerebral cortex after birth (Godin et al. 2011). Finally, recent studies by Taléns-Visconti and colleagues (2011) have demonstrated that ethanol affects the proliferation of neural progenitor cells and markedly reduces their potential to differentiate into mature neurons, astrocytes, and another type of glial cell called oligodendrocytes. Given this broad-spectrum impediment to nearly every neuronal developmental fate, it is possible that the observed impact of ethanol on the overall architecture and size of the brain in FAS children stems from effects on some aspect of PcG/TrxG regulation of neural precursor differentiation. Using a neurosphere model of differentiation, Mo and colleagues (2012) recently demonstrated that expression of the *Pax6* gene at a site other than where it usually is expressed could ameliorate the impact of ethanol on cell proliferation and neurogenesis. These results suggest that within a limited scope it may be possible to reverse alcohol’s effects on developmental programs.

## Conclusions

One of the most difficult aspects in the study of FASDs has been trying to explain the wide range of severity and enormous variation in FASD-associated birth defects. The process of organ formation is initiated during the early stages of embryonic development, and different rudimentary organ systems are formed and grow during unique developmental windows (Zorn and Wells 2009). Each organ system cycles between periods of intense growth and steady-state maintenance. The periods of growth are characterized by carefully orchestrated changes in DNA methylation and chromatin structure as differentiating cells are programmed with their epigenetic identity (Zhou et al. 2011). Studies using animal models analyzing the correlation of ethanol exposure at varying developmental time points with major periods of tissue growth strongly indicate that different tissues primarily are susceptible to ethanol-induced teratogenesis during specific developmental windows (Becker et al. 1996). Given the demonstrated ability of alcohol to alter DNA methylation and chromatin structure, it is likely that in organ systems which enter or are in a period of active epigenetic programming, ethanol exposure induces lasting epigenetic lesions that persist throughout organogenesis, whereas non-developing systems remain largely refractory to alcohol’s effects. Thus, the epigenetic errors resulting from alcohol exposure can vary greatly depending on the specific timing and dose of alcohol exposure, which can explain the wide diversity in severity and range of birth defects that characterize FASD (Becker et al. 1996).

Since their discovery, the PcG and TrxG protein complexes have been identified in numerous disease contexts, including cellular transformation of normal cells into tumor cells as well as structural defects and mental illness (Huang et al. 2007; Varambally et al. 2002; Yu et al. 1995). These studies have demonstrated that a molecular event or teratogen (e.g., ethanol) that alters PcG/ TrxG programming within even a few neural progenitor stem cells during fetal growth can disproportionately influence subsequent brain development and potentially impart severe neurological birth defects (Boyer et al. 2006, Hirabayashi and Gotch. 2010). A complete characterization of the involvement of PcG and TrxG complexes in the etiology of FASD will undoubtedly aid in understanding the role of epigenetic programming in this complex disorder.

## Figures and Tables

**Figure 1A:**
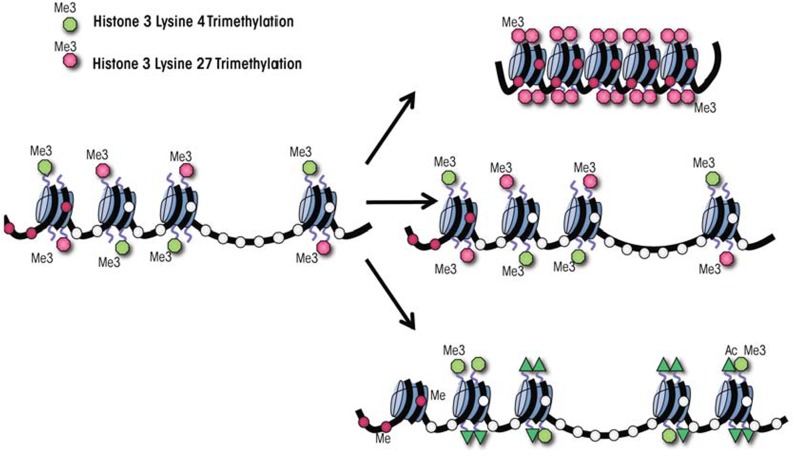
Bivalent state of the DNA in mammalian cells and its resolution during differentiation. In stem or progenitor cells, numerous developmentally relevant genes encoding factors that drive lineage-specific patterning are simultaneously marked with both activating and repressive histone modifications. This bivalent chromatin signature is thought to silence lineage-specifying genes through histone 3 lysine 27 trimethylation (H3K27me3) while at the same time poising them for activation during differentiation through the presence of histone 3 lysine 4 trimethylation (H3K4me3). As differentiation progresses, these domains can either adopt a silent conformation (top), become transcriptionally active (bottom), or persist into the next progenitor cell type (middle).

**Figure 1B:**
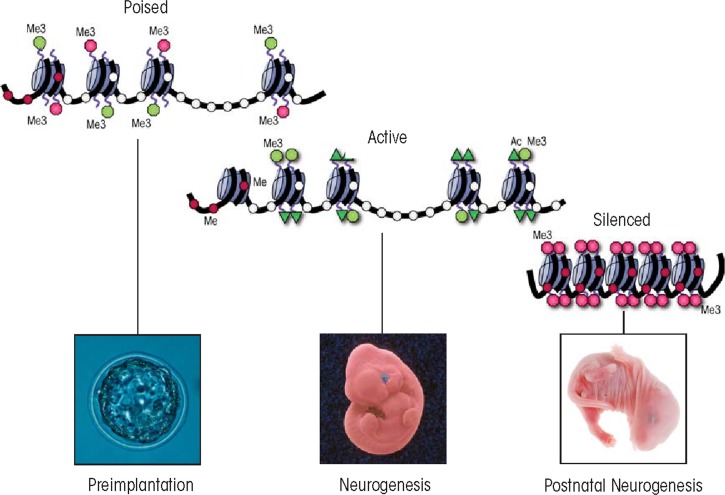
During development of the nervous system, many genes controlling neural patterning are held in a poised or bivalent conformation during early embryogenesis, resolve towards the active conformation during neural patterning, and are silenced during postnatal life. Repression (i.e., trimethylation of histone 3 lysine 27 [H3K27me3]) is imposed by the polycomb group proteins (PcG) (small red circles), whereas activation H3K4me3 is imparted by the mammalian homologues of the trithorax group proteins (TrxG) (green triangles). Correct biochemical function of these proteins and the coordination of the marks they impart are essential to mammalian neurogenesis.

## Footnotes

1 Stem cells still have the ability to differentiate into any type of specialized cell (e.g., nerve, blood, or muscle cells); therefore, they are called pluripotent. Progenitor cells already are more specialized and therefore committed to a certain type of tissue; for example, neural progenitor cells can develop into different types of nerve cells or supporting brain cells (i.e., glial cells) but can no longer differentiate into muscle or blood cells. Thus, these progenitor cells are multipotent but not pluripotent.

2 The term “facultative heterochromatin” refers to gene-rich regions of the genome that are silent, but which can dynamically cycle into periods of transcriptional activity. For example, a chromosome region containing a gene that is active only during late development will be silent during early development, become transcriptionally active during late development, and then return to a silent state for the adult stage of life. Conversely, other genomic regions are held perpetually in a tightly compact, silent state; these regions are known as constitutive heterochromatin.
